# Cytokine Dynamics in Acute Pancreatitis: The Quest for Biomarkers from Acute Disease to Disease Resolution

**DOI:** 10.3390/jcm13082287

**Published:** 2024-04-15

**Authors:** Filipa Malheiro, Miguel Ângelo-Dias, Teresa Lopes, Catarina Gregório Martins, Luis Miguel Borrego

**Affiliations:** 1 Internal Medicine Department, LUZ SAÚDE, Hospital da Luz Lisboa, 1500-650 Lisboa, Portugal; 2 CHRC, NOVA Medical School, Faculdade de Ciências Médicas, NMS, FCM, Universidade Nova de Lisboa, 1169-056 Lisboa, Portugal; miguel.dias@nms.unl.pt (M.Â.-D.); maria.lopes@nms.unl.pt (T.L.); catarina.martins@nms.unl.pt (C.G.M.); luis.borrego@nms.unl.pt (L.M.B.); 3 Immunology Department, NOVA Medical School, Faculdade de Ciências Médicas, NMS, FCM, Universidade Nova de Lisboa, 1169-056 Lisboa, Portugal; 4 Immunoallergy Department, LUZ SAÚDE, Hospital da Luz Lisboa, 1500-650 Lisboa, Portugal

**Keywords:** acute pancreatitis, severity, cytokines, vascular endothelial growth factor, epidermal growth factor, monocyte chemoattractant protein-1, IL-6, biomarker

## Abstract

**Background**: Acute pancreatitis (AP) is an inflammatory disease of the pancreas with incompletely known pathogenic mechanisms. This study aimed to explore the temporal changes in serum cytokines in patients with AP and to assess the association of these changes with disease severity. **Methods**: Fifty patients hospitalized with AP were enrolled, and their serum cytokine levels were analyzed at four different time points. A healthy control (HC) group of 30 outpatients was included. **Results**: AP patients showed increased levels of interleukin (IL)-6, IL-8, IL-10, vascular endothelial growth factor (VEGF), tumor necrosis factor (TNF)-alpha, and monocyte chemoattractant protein (MCP)-1 at admission when compared with HC. IL-6, VEGF, and EGF remained elevated 1 month after hospitalization and 6 months after discharge. Conclusions the Bedside Index of Severity in Acute Pancreatitis (BISAP) and severity classification of the revised Atlanta classification system, IL-6 and VEGF, determined 48 h after hospitalization, were the two cytokines consistently elevated in the most severe patients. Increased levels of IL-4, IL-6, IL-10, and TNF-alpha at admission and MCP-1 48 h after admission are also related to the length of hospital stay. **Conclusions**: Our study highlights the role cytokines play in the pathogenesis of AP and can be useful in the development of future drug trials for AP.

## 1. Introduction

Acute pancreatitis (AP) is an inflammatory disease of the pancreas with dysregulation of the immune system that can lead to multiple organ failure and death. There has been an increasing incidence of AP in developed countries [1]. Initially, there is injury or disruption of the pancreatic acini, which permits the leakage of pancreatic enzymes into pancreatic tissue. The leaked enzymes become activated in pancreatic tissue, initiating autodigestion and acute pancreatitis [2]. These events can be triggered by common acinar cell toxins like bile acids, alcohol, or nicotine as well as intraductal injury, like trauma or endoscopic retrograde cholangiopancreatography (ERCP) [3]. Injured acinar cells release chemokines, cytokines, and various adhesion molecules that recruit and mediate the infiltration of immune cells into the site of injury [4]. When immune cells infiltrate the pancreas, the cellular contents released from necrotic and injured cells activate monocytes and neutrophils that further activate inflammation. The production of pro-inflammatory cytokines including tumor necrosis factor (TNF), interleukin (IL)-1β, IL-6, and IL-18, among others, is amplified [5]. This immunological amplification of initial inflammation is due to a generalized cytokine-mediated hyperinflammatory response [6]. When these pro-inflammatory cytokines are released into the blood, the inflammation is no longer confined to the pancreas, and consequently, systemic inflammatory response syndrome (SIRS) develops [7]. Higher serum levels of TNF-α and IL-6, inducing more lymphocyte activation, have been associated with increased AP severity and organ dysfunction [8,9]. Very few studies have addressed the role of epidermal growth factor (EGF) and vascular endothelial growth factor (VEGF) in AP patients. A study in rats reported VEGF as an important factor in the pathogenesis of pancreatitis and severe cases of AP by causing edema and hemorrhage [10]. Although the determination of cytokines in the peripheral blood of patients with AP has been extensively reported, several limitations have been encountered in their interpretation. For instance, the use of different techniques, but also different time points for patient recruitment and for follow-up, limit the interpretation of data previously published [11]. Thus, a comprehensive characterization of inflammatory and anti-inflammatory cytokines at different time points in the evolution of AP might not only be of help for a better understanding of disease pathogenesis but also be helpful in finding much-needed new severity biomarkers in AP and aiding in the design of clinical trials with anticytokine therapies. Therefore, we aimed to explore the dynamics of cytokines in AP patients and assess potential associations between their changes and disease severity, from the early phases of AP until the healing process and full recovery from the disease.

## 2. Materials and Methods

### 2.1. Subjects and Sample Collection

Fifty AP patients (n = 50) admitted to Hospital da Luz Lisboa between February 2021 and March 2023 were consecutively recruited for this prospective observational study. The cohort of this study has been previously reported and published [12]. The diagnosis of AP was made according to the revised Atlanta classification system, which requires at least 2 of the following 3 features: abdominal pain characteristic of AP, serum amylase and/or lipase at least three times greater than the reference limit, and findings characteristic of acute pancreatitis on abdominal computerized tomography scan (CT scan) or transabdominal ultrasonography [13]. Only patients with onset of abdominal pain less than 48 h at hospital admission were included. Patients hospitalized for AP in the last 6 months, pregnant women, patients with important uncontrolled comorbidities such as organ disease (cardiac, renal, hepatic) and terminal neoplasms, patients on immunosuppressive or chemotherapy, and patients younger than 18 years or older than 85 years old were excluded. Patients were further classified according to the cause of AP, and according to severity as defined by the revised Atlanta classification system: mild acute pancreatitis (MAP), moderately severe acute pancreatitis (MSAP), or severe acute pancreatitis (SAP) [13]. The Bedside Index of Severity in Acute Pancreatitis (BISAP) score was also applied at admission to all patients [14]. AP clinical characteristics and outcomes such as cause and severity of AP, BISAP score, mechanical ventilation, ICU and hospital length of stay, complications during hospitalization, and mortality were also recorded.

Thirty age- and sex-matched healthy individuals (n = 30) were included as the healthy control group (HC). HC were ambulatory individuals observed at Hospital da Luz Lisboa, without previous pancreatic pathology or acute systemic disease.

Clinical and demographic data were analyzed in AP and HC groups, including gender, age, body mass index (BMI), and comorbidities. Additionally, data on other markers were collected from the hospital patient file, including C-reactive protein (CRP) and complete blood count. Blood samples were collected from patients with acute pancreatitis in the first 24 h of diagnosis (T1), at 48 h (T2) of hospitalization, 1 month after discharge (T3), and at least 6 months after discharge (T4). Three patients from the cohort study failed to attend the scheduled appointment (one at T3 and two at T4) but were still included in the analysis. One patient had just been diagnosed with squamous cancer and had started chemotherapy and three others decided to drop out of the study. The HC group had only one blood collection time point performed after recruitment. In all cases, venous blood was drawn from the antecubital vein and transferred to tubes without anti-coagulant, kept at room temperature for 30–120 min, centrifuged for 10 min, and the resulting serum was stored at −80 °C.

This study was approved by Hospital da Luz (CES/24/2020/ME) and NOVA Medical School (14/2019/ADENDA/CEFCM) ethics committees. Written informed consent was obtained from each subject before sample collection.

The serum samples were analyzed for 12 cytokines (i.e., IL-2, IL-4, IL-6, IL-8, IL-10, VEGF, interferon (IFN)-gamma, TNF-alpha, IL-1alpha, IL-1beta, monocyte chemoattractant protein (MCP-1), and EGF) included in the Cytokine and Growth Factors Array (Randox Laboratories Ltd., Crumlin, Northern Ireland, United Kingdom), using Multiplex Biochip Array Technology. The protocol followed the manufacturer’s instructions, and the biochips were read in an Evidence Investigator^TM^ analyzer (also from Randox Laboratories Ltd.). Samples were run in single replicates, with all samples from the same patient assayed in the same run. To assure reproducibility, quality control samples (Randox Cytokine Multianalyte Controls, levels I, II, and III) were assayed in all runs, and their values were within the acceptable ranges defined. Patient samples with values above the upper limit of quantification (ULOQ) were replaced by ULOQ, and non-detectable concentrations (<LOD) were discarded. Cytokines with more than 70% non-detectable values were not considered.

### 2.2. Statistical Analysis

Categorical variables were presented as absolute frequencies and percentages, and associations between them were analyzed with Fisher’s exact test.

CRP and cytokine concentrations were Log_10_-transformed to improve the normality of the residuals and data visualization. The normality of the data was assessed by visual inspection through QQ plots of the residuals and by using the D’Agostino–Pearson normality test when necessary.

A mixed-effects model with Geisser–Greenhouse correction was used to explore the time-dependent development of individual cytokines and CRP. The serum concentration of each cytokine was Log_10_-transformed and modeled with time point as a fixed effect and patient as a random effect. Multiple comparisons between time points were performed with Tukey’s multiple comparisons test. Comparisons between the control group and each AP time point were performed with Brown–Forsythe and Welch ANOVA tests followed by Dunnett’s T3 multiple comparisons test; otherwise, the non-parametric Kruskal–Wallis test followed by Dunn’s multiple comparisons tests were used. All the analyses described above were performed after outlier exclusion using the ROUT method, as recommended by GraphPad, using a Q value of 0.2%. Two-group analyses of unpaired normally distributed data were performed with the Unpaired t-test or Unpaired t-test with Welch’s correction, as appropriate.

Correlations between Log-transformed cytokine concentrations were calculated separately within each time point using Pearson’s correlation coefficient. Correlations between length of stay at the hospital (LOS) were calculated using Spearman’s correlation coefficient. Each test used is indicated in the respective figure and table legends.

For all analyses, a *p*-value of less than 0.05 was considered significant: * *p* < 0.05, ** *p* < 0.01, *** *p* < 0.001, **** *p* < 0.0001.

Statistical analyses were performed by using GraphPad Prism v10.1.2 for Windows (GraphPad Software, Boston, MA, USA, www.graphpad.com). Visualizations were made with GraphPad Prism v10.1.2 (GraphPad Software, Boston, MA, USA, www.graphpad.com), and Instant Clue v0.11.3 software for Windows [15].

## 3. Results

Baseline characteristics of patients with AP and HC are summarized in Table 1. Most cases were caused by gallstones, and the mean length of stay at the hospital was 6 days. Sixteen patients (32%) scored a BISAP of 2 or 3.

The cytokines IL-2, IFN-gamma, and IL-1beta had high proportions of non-detectable values in the multiplex assay and were excluded from the analysis (Supplementary Materials Table S1). The remaining nine cytokines were within the detection limits in at least 55% of the serum samples. Six cytokines (IL-6, IL-8, VEGF, TNF-alpha, MCP-1, and EGF) had detectable serum concentrations in more than 89% of total samples, while three cytokines (IL-4, IL-10, and IL-1alpha) had detectable serum concentrations in more than 55% of total samples. The proportion of detectable cytokines was generally higher in T1, decreasing afterward to the lowest value in T4 and controls.

### 3.1. Serum Concentrations for Evaluated Cytokines

**Figure 1:**
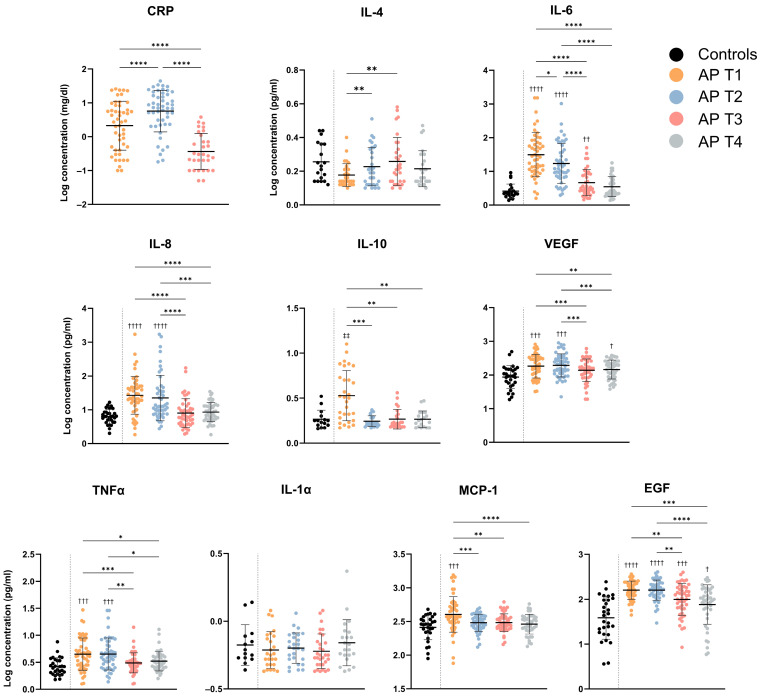
Serum concentration of CRP (mg/dL) and cytokines (pg/mL) in controls and AP T1, T2, T3, and T4 assessments. Data are reported as mean ± SD of Log_10_ concentration. Detailed results are presented in Table 2. Differences between AP time points (*) were tested with mixed-effects model followed by Tukey’s multiple comparisons test: * *p* < 0.05; ** *p* < 0.01; *** *p* < 0.001; **** *p* < 0.0001. Symbols at the top of each AP timepoint indicate statistically significant differences compared to controls (^†^ and ^‡^). Comparisons between controls (^†^) were tested with Brown–Forsythe and Welch ANOVA tests followed by Dunnett’s T3 multiple comparisons test: ^†^ *p* < 0.05; ^††^ *p* < 0.01; ^†††^ *p* < 0.001; ^††††^ *p* < 0.0001. Comparisons between controls regarding IL-10 and IL-4 (^‡‡^) were tested with the Kruskal–Wallis test followed by Dunn’s multiple comparisons tests: ^‡‡^ *p* < 0.01. CRP—C-reactive protein; EGF—endothelial growth factor; IL—interleukin; INF—interferon; VEGF—vascular endothelial growth factor; TNF—tumor necrosis factor; MCP—monocyte chemotactic protein.

The serum cytokine values observed in the healthy controls and along the four time points of the AP patients’ follow-up are presented in Table 2. The evolution in mean cytokine concentrations for the nine cytokines with analyzable values is shown in Figure 1. Changes in serum cytokine concentrations during 6 months after discharge from AP follow-up were observed, with major differences observed during the acute phase (T1 and T2). Cytokine concentrations at T3, and especially at T4, reflect the resolution of the inflammatory boost observed during the acute phase and were, in general, not significantly different from the concentrations observed in healthy controls. Exceptions were IL-6, VEGF, and EGF.

For some cytokines, i.e., IL-6 or IL-10, notable variations between individuals were evident, as shown by the relatively large standard deviations and ranges observed. A great span in absolute concentrations for different cytokines was also found, with IL-8 having the highest quantifiable value (≥1679 pg/mL) and IL-1alpha having the lowest detectable (0.43 pg/mL) concentration at T1.

Seven out of the nine cytokines evaluated were significantly increased in AP patients at T1, compared to HC, and decreased from T1 to T2 evaluations, including IL-6, IL-8, IL-10, VEGF, TNF-alpha, MCP-1, and EGF, while no differences were observed between T3 and T4 in terms of cytokine concentrations. Oppositely, IL-4 levels were lower at T1 compared to T2 and T3 evaluations, though not significantly different from controls. IL-1alpha levels were relatively stable during follow-up and were not different from the controls.

**Figure 2:**
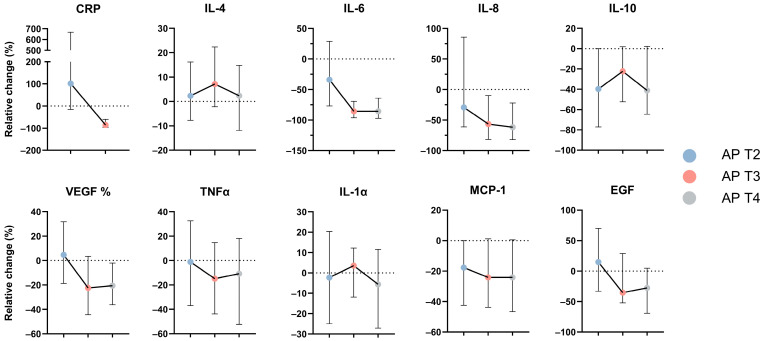
Relative change in serum concentration of CRP and cytokines at AP T2, T3, and T4 assessments. Data are reported as median with IQR of relative change (%) normalized to AP T1. CRP—C-reactive protein; EGF—endothelial growth factor; IL—interleukin; INF—interferon; VEGF—vascular endothelial growth factor; TNF—tumor necrosis factor; MCP—monocyte chemotactic protein.

To better assess the overall variations from acute disease to AP resolution and adjust for individual basal levels, the C-reactive protein (CRP) and cytokine values were normalized to the first study visit by calculating the relative change from the measurements at T1 (Figure 2). A general tendency for decreased cytokine concentrations after AP admission was observed and was particularly evident for IL-6 and IL-8, which presented the greatest median variation between the four time points during AP follow-up, decreasing more than 50% (more than 75% for IL-6) from their initial concentration at T1 to T4. Within those cytokines that significantly differ along the follow-up of AP patients, IL-4 was the one with the lowest relative change, with less than 10% variation from T1 to T3 and T4. The timing and magnitude of the decrease also varied. While IL-6, IL-8, IL-10, and MCP-1 showed an evident decrease at T2 (between 18% and 40% of their initial concentrations), VEGF, TNF-alpha, and EGF were relatively stable from T1 to T2 (variations from −1% to +14%). Curiously, IL-10 was the cytokine with greater variation at T2 (−40%), though with a large IQR interval. The concentrations of CRP presented the greatest variations. Indeed, CRP duplicated from T1 to T2 (102%) and then dramatically decreased at T3 showing a negative relative change of 85% from T1 to T3.

### 3.2. Cytokine Correlation Analyses in AP Patients and Controls

**Figure 3:**
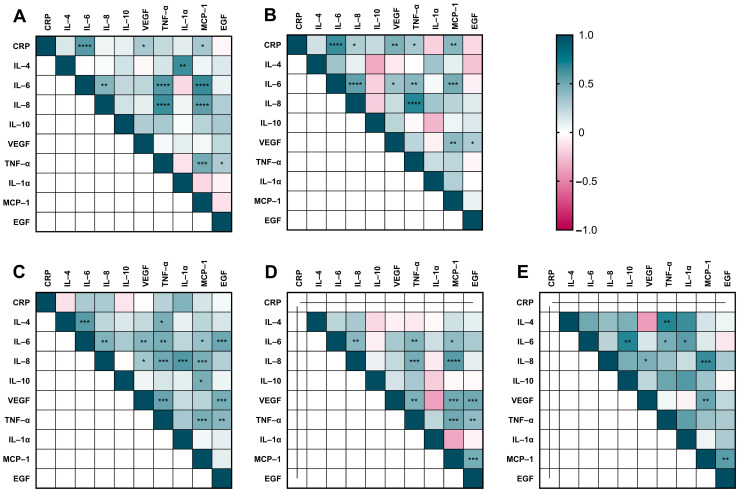
Pearson correlation heatmaps of CRP and serum cytokines. AP at T1 (**A**), T2 (**B**), T3 (**C**), and T4 (**D**), and controls (**E**). The correlation coefficient (r) is shown with a double gradient intensity scale, and statistical significance for a given pair is indicated. * *p* ≤ 0.05; ** *p* ≤ 0.01; *** *p* ≤ 0.001; **** *p* ≤ 0.0001.

The assessment of associations between cytokines at all time points is depicted in Figure 3. Of a total of 207 possible cytokine pairs considering the four AP time points and the controls, 57 pairs showed significant correlations. Each time point showed a different correlation pattern, as presented in the respective heatmaps, and only positive correlations with statistical significance were found (both in AP time points and in control patients), mainly between the serum levels of CRP, IL-6, IL-8, TNF-alpha, and MCP-1.

The highest correlations observed (within those with statistical significance) were between TNF-alpha and IL-8 (r = 0.683; AP T2), IL-6 and MCP-1 (r = 0.654; AP T1), and IL-6 and CRP (r = 0.644; AP T2). When calculating the mean correlation of possible cytokine pairs combining the four AP time points evaluated, the pairs with the highest mean correlation were IL-8 and TNF-α (r = 0.573), IL-6 and CRP (r = 0.504; no T4 considered), and TNF-alpha and IL-6 (r = 0.472).

**Figure 4:**
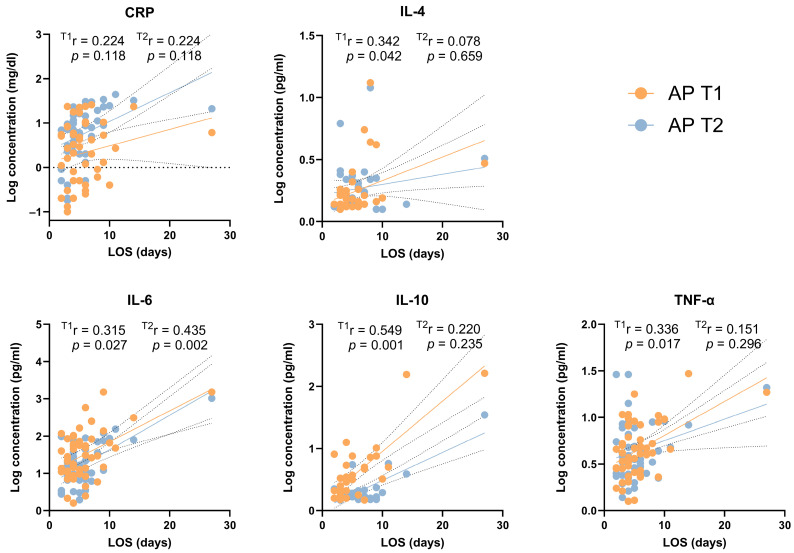
Spearman’s correlation of LOS with serum cytokine in AP patients at T1 and T2. The correlation coefficients, 95% confidence intervals (dashed lines), and *p*-values are displayed for each group. AP, acute pancreatitis; LOS, length of stay at hospital; CRP—C-reactive protein; IL—interleukin; TNF—tumor necrosis factor.

The evaluation of associations between CRP and cytokine concentrations at T1 and age, BMI, or LOS revealed significant results (Figure 4). The levels of IL-10 and TNF-alpha at T1 showed positive correlations with age (r ≥ 0.298, *p* ≤ 0.036), while IL-1alpha was negatively correlated with this parameter (r = −0.519; *p* = 0.005). CRP levels at T1 were positively correlated with BMI (r = 0.302, *p* = 0.033), while IL-4, IL-6, IL-10, and TNF-alpha levels at T1 were positively correlated with LOS. At T2, IL-6 levels were still positively correlated with LOS, as well as CRP, and MCP-1 (Figure 4).

### 3.3. Cytokine Concentrations According to Severity and Clinical Characteristics

**Figure 5:**
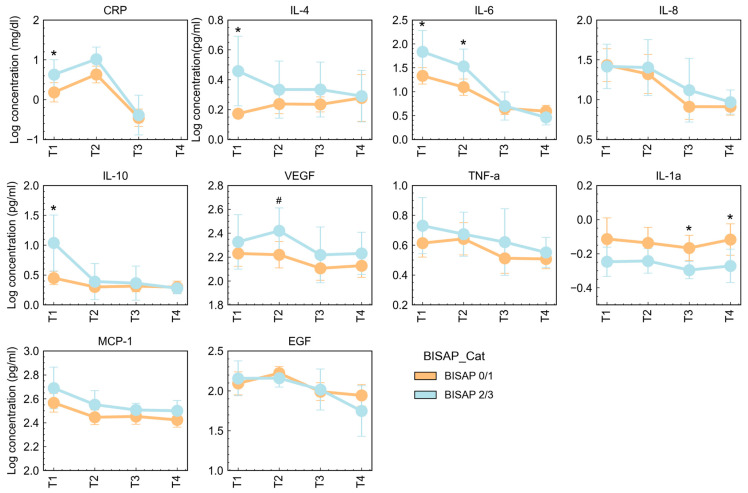
Serum concentration of CRP (mg/dL) and cytokines (pg/mL) in AP T1, T2, T3, and T4 assessments, according to BISAP classification. Data are reported as mean ± SD. Differences between BISAP subgroups at each AP time point were tested using Unpaired *t*-test with (*) or without (^#^) Welch’s correction: * *p* < 0.05. CRP—C-reactive protein; EGF—endothelial growth factor; IL—interleukin; INF—interferon; VEGF—vascular endothelial growth factor; TNF—tumor necrosis factor; MCP—monocyte chemotactic protein.

The changes in cytokine levels were not independent of BISAP classification, disease severity, BMI group, and cause of AP. Overall, cytokine levels were increased in patients with higher BISAP scores, especially at admission (Figure 5). Patients with a BISAP classification of 2 or 3 (BISAP 2/3) showed increased concentrations of CRP (*p* = 0.043), IL-4 (*p* = 0.022), IL-6 (*p* = 0.037), and IL-10 (*p* = 0.020) at admission (T1), and increased concentrations of VEGF (*p* = 0.045) at T2 compared to patients with a BISAP classification of 0 or 1 (BISAP 0/1). The higher levels of IL-6 were kept at T2 in patients with BISAP 2/3 (*p* = 0.038). With the exception of IL-1alpha, which maintained higher concentrations in patients with lower BISAP 0/1 (*p* ≤ 0.036), no differences were observed at T3 and T4 comparing BISAP subgroups.

**Figure 6:**
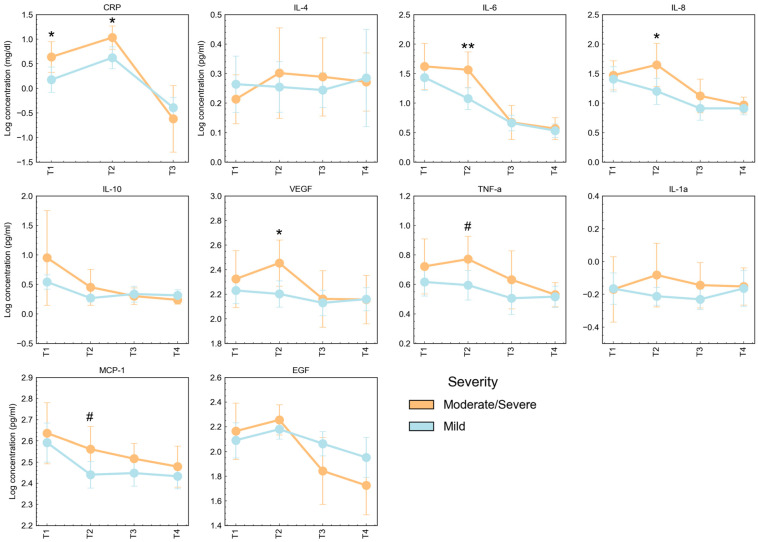
Serum concentration of CRP (mg/dL) and cytokines (pg/mL) in AP T1, T2, T3, and T4 assessments, according to the severity of the Atlanta classification system. Data are reported as mean ± SD. Differences between BISAP subgroups at each AP time point were tested using Unpaired t-test with (*) or without (^#^) Welch’s correction: * *p* < 0.05; ** *p* < 0.01. CRP—C-reactive protein; EGF—endothelial growth factor; IL—interleukin; INF—interferon; VEGF—vascular endothelial growth factor; TNF—tumor necrosis factor; MCP—monocyte chemotactic protein.

Patients with different severity presentations of the disease showed different evolutions in cytokine concentration (Figure 6). Patients with MSAP to SAP presented at admission with higher CRP levels (*p* = 0.022), but similar concentration levels of cytokines. However, after T1, while MAP patients did not present significant changes along follow-up (T2 to T4), MSAP and SAP patients had significantly higher levels of many biomarkers, namely, CRP (*p* = 0.012), IL-6 (*p* = 0.008), IL-8 (*p* = 0.036), VEGF (*p* = 0.022), TNF-alpha (*p* = 0.049), and MCP-1 (*p* = 0.042). Concentration levels were similar at T3 and T4 for all cytokines. IL-6 concentration in MAP patients showed a subtle decrease from T1 to T4, while in MSAP and SAP patients, the levels of IL-6 were stable from T1 to T2, followed by a marked decrease at T3.

The etiology of AP, whether due to gallstone or other, was also associated with changes in cytokine concentration, namely, IL-8 and TNF-alpha, which showed increased levels at T1 in patients with gallstone as the AP cause (Supplementary Materials Figure S1). Aside from the higher CRP levels observed at T2 of AP patients with obesity (compared to overweight patients), no other significant differences were observed considering subgroups according to BMI (Supplementary Materials Figure S2). The changes in cytokine patterns were independent of patient sex.

## 4. Discussion

Many of the mechanisms responsible for the severity and evolution of AP, including those involving the immune system, are still unknown, and this is why it is still so difficult to identify and treat patients with acute pancreatitis, especially in the most severe cases.

Cytokines are soluble proteins with low molecular weights, produced and secreted from a variety of cells including lymphocytes, macrophages, natural killer cells, mast cells, and stromal cells. They are important mediators associated with the communication network of the immune system [16]. Several cytokines and chemokines have been associated with AP severity, especially at patient admission [17,18]. There have been contradictory studies on whether cytokines can predict the severity of AP in patients, with IL-6, IL-8, and IL-10 being the most studied cytokines [18,19,20]. Using a panel or a combination of cytokines to study the relationship with AP severity has also been reported [21,22]. Also, the best timing to determine cytokine levels is far from consensual, as some studies show the peak levels of cytokines at admission, while others show that these peaks occur after the first 24 h of hospitalization [17,21,23,24]. Among other markers, most physicians and guidelines consider CRP at 48 h after symptom onset as the best indicator for assessing disease severity [23]. In our study, we observed a decrease in the levels of some markers from T1 to T2, namely, CRP, IL-4, IL-6, IL-10, and MCP-1. Furthermore, we observed differences between AP patients at T1 and HC, again in IL-6, IL-10, and MCP-1, but also in IL-8, VEGF, and TNF-alpha. As expected, the decrease in the levels of most studied cytokines at T3 and T4, in comparison to the levels during the acute phase, along with the absence of significant differences from the levels observed in HC, likely indicates the resolution of AP, which is evident as early as one month after hospital admission. The exceptions were VEGF and EGF, which still showed differences at T4 between AP patients and HC.

Proinflammatory cytokines have been widely studied in the context of AP, but much less attention has been paid to growth factors at AP onset or during the healing course of the disease. In the present study, we identified increased EGF and VEGF at different time points in AP patients. EGF was one of the first discovered growth factors and plays a central role in regulating cell proliferation and differentiation [25]. EGF has a role in cancer progression, as its tyrosine kinase activity is responsible for tumor survival, growth, and metastasis. The EGF receptor is expressed in a variety of human tissues including most epithelial tissues, fibroblasts, and endothelial cells, meaning that EGF plays a key role in wound healing and in maintaining tissue integrity [26,27]. We admit that EGF might still play a role 6 months after diagnosis of AP, probably due to its role in tissue healing.

Ueda et al. described that VEGF levels were higher in patients with the most severe AP and speculated that VEGF does not function as a vascular permeability factor but as a protective factor by its anti-apoptotic effect against organ injuries in AP [28]. VEGF has also been studied in samples of pancreatic tissue from patients with AP, and it was admitted that this vascular growth factor can play an important role in tracking the evolution and pathology of acute pancreatitis [29]. VEGFs are regulators of angiogenesis expressed in response to soluble mediators, such as cytokines and growth factors. Their main functions include blood vessel formation, the regulation of vascular permeability, stem cell, monocyte, and macrophage recruitment, as well as the maintenance of bone homeostasis and repair. Angiogenesis has a pivotal role in chronic pathologic conditions, such as tumorigenesis, inflammatory immune diseases, and bone loss [30]. VEGF may play an important role in the pathogenesis of pancreatitis by causing edema and hemorrhage in SAP in the early stages of AP, while its elevation may reflect the severity of pancreatic injury [10]. The inhibition of VEGF or its receptor signaling system has been used to treat several types of cancer, such as colorectal cancer, renal cell carcinoma, and non-small-cell lung carcinoma [31]. The inhibition of VEGF receptor signaling is also used to treat inflammatory diseases in which angiogenesis plays a significant role, such as rheumatoid arthritis and psoriasis [32,33]. For these reasons, the inhibition of VEGF or its receptor signaling system might be an attractive target for therapeutic intervention in acute pancreatitis. The reason why VEGF and EGF were the only measured cytokines that remained elevated at T3 and T4 compared to HC is unclear. We hypothesize that it might be related to the healing of the inflammatory process, which could potentially take longer than expected in AP patients.

Considering that some authors have highlighted the disadvantage of using a single biomarker for AP severity prediction, it might be of interest to study the association between individual cytokines at different time points. Not surprisingly, T1 and T2 showed the strongest positive correlations with statistical significance between cytokine pairs namely, IL-6 and MCP-1 at T1, IL-6 and MCP-1 at T2, and IL-6 and CRP at T2. The primary sources of MCP-1 are epithelial cells, endothelial cells, smooth muscle cells, monocytes and macrophages, fibroblasts, astrocytes, and microglial cells. A study by Papachristou et al. reported that MCP-1 serum levels, measured during the first 24 h of diagnosis of AP, appear to be an accurate predictor of the severity of acute pancreatitis and death [34]. MCP-1 is an important chemokine that plays a fundamental role in several pathological conditions, such as cardiovascular diseases, brain pathologies, bone and joint disorders, respiratory infections, cancer, endothelial dysfunction, and, recently, in the COVID-19 pandemic, by activating the signaling pathways regulating cell migration [35]. However, MCP-1 is only one of several chemokines upregulated in acute pancreatitis, and evidence of its pathogenic role is still not known. Full knowledge of the role of MCP-1 in AP might also be another target for the development of new drugs in AP.

Acute pancreatitis is a high-cost disease, with a median cost of nearly 7000 USD per hospitalization in 2013 [36]. Few studies have reported relationships between cytokine levels and length of hospital stay, even though AP constitutes a high economic burden, and hospitalizations due to AP have been increasing in recent decades [37]. In our findings, we observed that at T1, higher levels of IL-4, IL6, IL-10, and TNF-alpha were associated with a longer length of hospital stay. Additionally, at T2, elevated levels of IL-6, CRP, and MCP-1 were also associated with longer periods in the hospital.

When considering the BISAP severity score used in clinical practice, we found that IL-4, IL-6, and IL-10 levels at T1 and VEGF and IL-6 levels at T2 were increased in patients with higher BISAP scores (≥2), despite the fact that no significant differences were observed considering different BISAP scores at T3 and T4

Using the revised Atlanta classification system of AP severity of 2012, we observed increased IL-6, IL-8, VEGF, TNF-alpha, and MCP-1 cytokine levels at T2 in patients with moderate to severe AP. Interestingly, when considering both AP severity scores (BISAP and Atlanta), only IL-6 and VEGF levels consistently emerged as putative reliable markers of disease severity at T2, since both cytokines were elevated at this time point in patients with higher BISAP and Atlanta severity scores. When considering IL-6, these results come as no surprise, but there are very few studies that include VEGF and its role in acute pancreatitis patients; therefore, we can conclude that our study may constitute a milestone in the development of possible new markers for assessing the severity of AP [17,38].

We acknowledge some limitations of this study. First, we consider the small number of patients with severe disease as a main limitation. Most patients had a BISAP score of less than two or had mild to moderately severe AP. Due to the smaller sample size of severe cases, the study may not fully capture the characteristics and outcomes specific to patients with severe AP. This could include variations in cytokine levels and other factors that may be more pronounced in severe cases. Increasing the number of patients in this subgroup would certainly provide a more comprehensive understanding of cytokine dynamics and their role in severe AP, leading to improved diagnosis and management strategies. A wider range of cytokines might also be of interest, including local cytokine and chemokine levels derived from injured acinar cells and different inflammatory cell types that increase due to pancreatic injury. The inclusion of these markers in future studies may further help to understand the pathogenesis of AP.

Due to the variable course of AP, several predictive models have been developed to foresee the outcome of the disease. However, to date, no soluble predictor of AP severity can be reasonably used in clinical practice within 24 to 48 h of admission [39,40]. Our study results corroborate the biological relevance of inflammatory cytokines in the pathophysiology of AP. However, none of the already studied cytokines has arisen as an undisputable biomarker of AP severity. This study aimed to characterize the baseline levels of these cytokines and their trajectories over time, from the early course of AP until the healing process occurs, in a cohort of AP patients and also compared them to healthy controls.

Better knowledge of these variations over time and their relationship with AP severity is of great help in understanding the immune mechanisms responsible for AP and its severity. The treatment of immune-mediated inflammatory diseases has changed dramatically during the past 20 years owing to the approval of several monoclonal antibodies and fusion proteins that target inflammatory cytokines or their receptors [41]. Our findings also guide future studies in the search for new accessible markers of AP severity, such as VEGF, for which there are several drugs already approved and used in oncology, namely, monoclonal antibodies against VEGF.

## 5. Conclusions

Cytokine levels in the peripheral blood of patients with AP were related to the severity of this disease. New soluble markers of AP severity, such as VEGF, MCP-1, and EGF, emerged as valuable references for future studies. These markers may ultimately prove to be of utmost interest in the improvement of clinical outcomes and the reduction in complications and hospitalizations of patients with AP, as well as helping in the design of new therapeutic strategies for patients with AP.

## Figures and Tables

**Table 1:** Demographic characteristics of patients with AP and HC.

Characteristics	AP (n = 50)	HC (n = 30)	*p*-Value
Age, years, mean (SD)	59.9 (14.6)	58.1 (16.6)	n.s. ^a^
Gender, n (%)			n.s. ^b^
Male	23 (46)	14 (47)	
Female	27 (54)	16 (53)	
BMI, kg/m^2^, mean (SD)	29.0 (5.8)	26.3 (3.5)	n.s. ^a^
Cause (%)		-	-
Gallstone	23 (46)		
Alcoholic	5 (10)		
Unknown	21 (42)		
Other	1 (2)		
Severity, n (%)		-	-
Mild	34 (66)		
Moderate	13 (28)		
Severe	3 (6)		
30-day mortality, n (%)	0 (0)	-	-
ICU, n (%)	3 (6)	-	-
Mechanical ventilation, n (%)	1 (2)	-	-
BISAP score, n (0)		-	-
0	14 (28)		
1	20 (40)		
2	9 (18)		
3	7 (14)		
AP criteria, n (%)		-	-
2	26 (52)		
3	24 (48)		
LOS, days, median [IQR]	6 [3–6]	-	-

Abbreviations: AP—acute pancreatitis; HC—healthy control; BMI—body mass index; ICU—intensive care unit; LOS—length of stay at hospital; IQR—interquartile range; SD—standard deviation; n.s.—not significant. ^a^ Unpaired *t*-test with Welch’s correction; ^b^ Fisher’s exact test.

**Table 2:** Cytokines and CRP concentration levels in HC and its variation along follow-up in AP.

	HC	AP T1	AP T2	AP T3	AP T4	*p*-Value *
CRP	-	0.32 ± 0.72 (−1.00; 1.40)	0.75 ± 0.61 (−0.74; 1.6)	−0.44 ± 0.53 (−1.30; 0.58)	-	<0.0001
IL-4	0.25 ± 0.11 (0.12; 0.44)	0.18 ± 0.07 (0.10; 0.40)	0.23 ± 0.11 (0.1; 0.51)	0.26 ± 0.14 (0.10; 0.58)	0.21 ± 0.11 (0.10; 0.47)	0.013
IL-6	0.41 ± 0.21 (0.14; 0.96)	1.50 ± 0.66 (0.20; 3.2)	1.20 ± 0.59 (0.29; 3.00)	0.67 ± 0.39 (0.16; 1.70)	0.54 ± 0.30 (0.14; 1.30)	<0.0001
IL-8	0.80 ± 0.21 (0.30; 1.20)	1.40 ± 0.56 (0.26; 3.2)	1.30 ± 0.67 (0.44; 3.20)	0.90 ± 0.42 (0.28; 2.2)	0.93 ± 0.28 (0.26; 1.5)	<0.0001
IL-10	0.26 ± 0.10 (0.16; 0.52)	0.53 ± 0.28 (0.17; 1.10)	0.24 ± 0.06 (0.17; 0.37)	0.27 ± 0.11 (0.17; 0.56)	0.27 ± 0.09 (0.17; 0.47)	<0.0001
VEGF	1.90 ± 0.34 (1.30; 2.70)	2.30 ± 0.35 (1.50; 2.90)	2.30 ± 0.34 (1.40; 3.00)	2.10 ± 0.33 (1.30; 2.80)	2.20 ± 0.28 (1.60; 2.60)	<0.0001
TNF-α	0.42 ± 0.16 (0.18; 0.88)	0.65 ± 0.30 (0.10; 1.50)	0.65 ± 0.30 (0.14; 1.50)	0.49 ± 0.18 (0.10; 1.2)	0.52 ± 0.18 (0.20; 1.10)	0.0001
IL-1α	−0.18 ± 0.15 (−0.36; 0.14)	−0.21 ± 0.14 (−0.37; 0.08)	−0.20 ± 0.11 (−0.37; 0.06)	−0.22 ± 0.13 (−0.37; 0.08)	−0.16 ± 0.17 (−0.37; 0.37)	0.577
MCP-1	2.40 ± 0.18 (2.00; 2.70)	2.60 ± 0.26 (1.90; 3.20)	2.50 ± 0.13 (2.10; 2.70)	2.50 ± 0.13 (2.20; 2.80)	2.4 ± 0.17 (1.9; 2.8)	<0.0001
EGF	1.60 ± 0.47 (0.56; 2.40)	2.20 ± 0.20 (1.70; 2.60)	2.20 ± 0.23 (1.50; 2.60)	2.00 ± 0.35 (0.93; 2.60)	1.9 ± 0.44 (0.77; 2.5)	<0.0001

Cytokine (pg/mL) and CRP (mg/dL) data are reported as mean ± SD (min; max) of Log10 concentration. * *p*-value from mixed-effects model of AP time points. CRP—C-reactive protein; EGF—endothelial growth factor; IL—interleukin; HC—healthy controls; AP—acute pancreatitis; VEGF—vascular endothelial growth factor; TNF—tumor necrosis factor; MCP—monocyte stimulating factor.

## Data Availability

The data that support the findings of this study are available from the corresponding author upon reasonable request.

## Supplementary Materials

The following supporting information can be downloaded at: https://www.mdpi.com/article/10.3390/jcm13082287/s1, Table S1. Immunoassay performance; Figure S1: Serum concentration of CRP (mg/dL) and cytokines (pg/mL) in AP T1, T2, T3, and T4 assessments, according to cause of AP; Figure S2: Serum concentration of CRP (mg/dL) and cytokines (pg/mL) in AP T1, T2, T3, and T4 assessments, according to BMI classification.
