# Supplementary material for: Cytokine Dynamics in Acute Pancreatitis: The Quest for Biomarkers from Acute Disease to Disease Resolution
Source: J Clin Med. 2024 Apr 15;13(8):2287. doi: 10.3390/jcm13082287 (PMC11051017; doi:10.3390/jcm13082287)
Supplement: Supplementary file 1 [file jcm-13-02287-s001.zip › jcm-2903436-supplementary.pptx]

## Slide 1
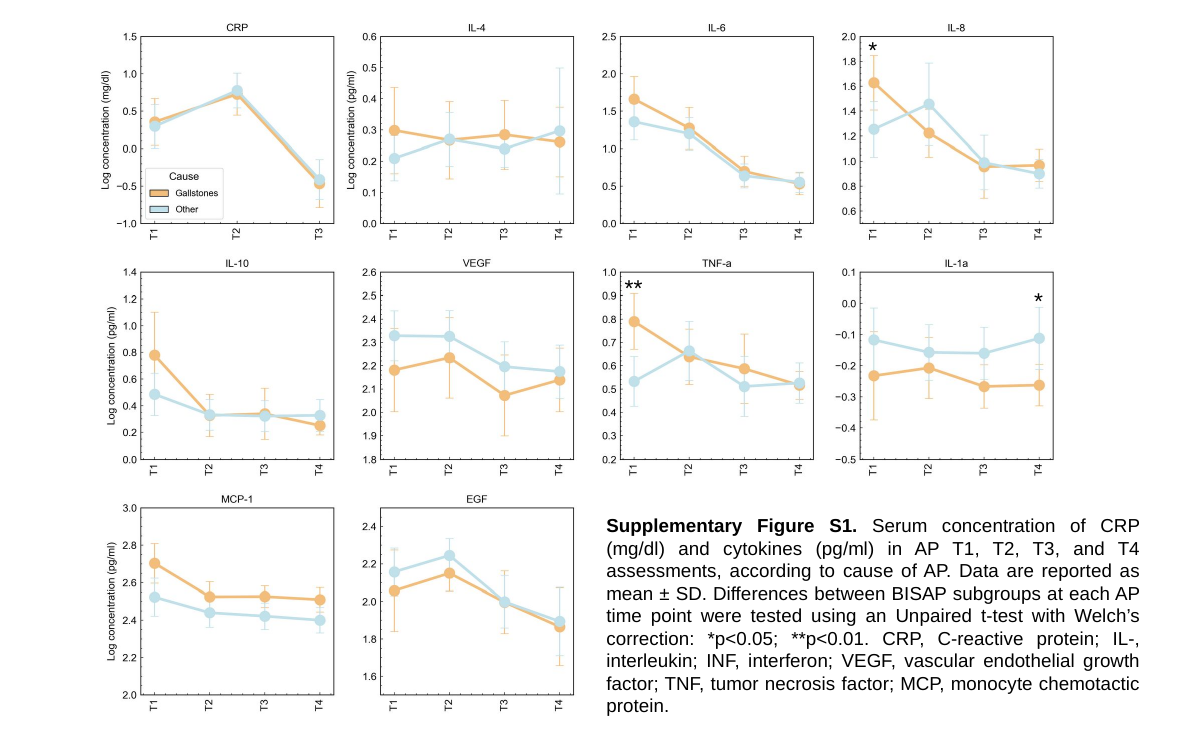

*
**
*
Supplementary Figure S1. Serum concentration of CRP (mg/dl) and cytokines (pg/ml) in AP T1, T2, T3, and T4 assessments, according to cause of AP. Data are reported as mean ± SD. Differences between BISAP subgroups at each AP time point were tested using an Unpaired t-test with Welch’s correction: *p<0.05; **p<0.01. CRP, C-reactive protein; IL-, interleukin; INF, interferon; VEGF, vascular endothelial growth factor; TNF, tumor necrosis factor; MCP, monocyte chemotactic protein.

## Slide 2
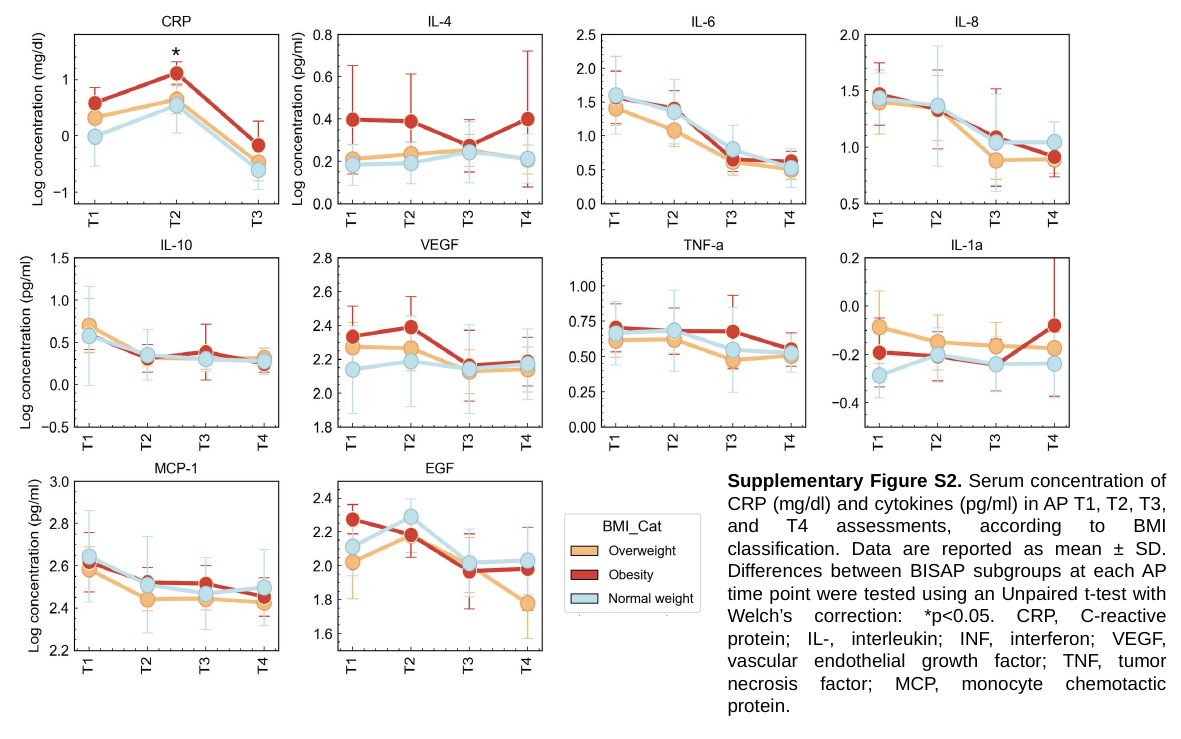

*
Supplementary Figure S2. Serum concentration of CRP (mg/dl) and cytokines (pg/ml) in AP T1, T2, T3, and T4 assessments, according to BMI classification. Data are reported as mean ± SD. Differences between BISAP subgroups at each AP time point were tested using an Unpaired t-test with Welch’s correction: *p<0.05. CRP, C-reactive protein; IL-, interleukin; INF, interferon; VEGF, vascular endothelial growth factor; TNF, tumor necrosis factor; MCP, monocyte chemotactic protein.
